# Chemoreceptors of *Escherichia coli* CFT073 Play Redundant Roles in Chemotaxis toward Urine

**DOI:** 10.1371/journal.pone.0054133

**Published:** 2013-01-30

**Authors:** Erica L. Raterman, Rodney A. Welch

**Affiliations:** Department of Medical Microbiology and Immunology, University of Wisconsin-Madison, Madison, Wisconsin, United States of America; University of Würzburg, Germany

## Abstract

Community-acquired urinary tract infections (UTIs) are commonly caused by uropathogenic *Escherichia coli* (UPEC). We hypothesize that chemotaxis toward ligands present in urine could direct UPEC into and up the urinary tract. Wild-type *E. coli* CFT073 and chemoreceptor mutants with *tsr*, *tar*, or *aer* deletions were tested for chemotaxis toward human urine in the capillary tube assay. Wild-type CFT073 was attracted toward urine, and Tsr and Tar were the chemoreceptors mainly responsible for mediating this response. The individual components of urine including L-amino acids, D-amino acids and various organic compounds were also tested in the capillary assay with wild-type CFT073. Our results indicate that CFT073 is attracted toward some L- amino acids and possibly toward some D-amino acids but not other common compounds found in urine such as urea, creatinine and glucuronic acid. In the murine model of UTI, the loss of any two chemoreceptors did not affect the ability of the bacteria to compete with the wild-type strain. Our data suggest that the presence of any strong attractant and its associated chemoreceptor might be sufficient for colonization of the urinary tract and that amino acids are the main chemoattractants for *E. coli* strain CFT073 in this niche.

## Introduction

Urinary tract infections (UTIs) are some of the most commonly diagnosed and treated infections in the United States with 40–60% of women experiencing at least one UTI in their lifetime [1], [2]. Most community-acquired UTIs are caused by uropathogenic *Escherichia coli* (UPEC) [3]. UPEC likely gain access to the urinary tract when the urethra is contaminated with intestinal microorganisms. After colonization of the urethra, the bacteria ascend the urethra to the bladder and, in some cases, continue up the ureters to the kidneys [4]. Known UPEC virulence and fitness factors include type I fimbriae, Pap pili, flagella, hemolysin, iron acquisition systems and toxins [3], [5]–[7], though factors important for early ascension of the urethra and the ureters and subsequent colonization of the bladder and kidneys remain largely unidentified. Previous reports indicate that motility is important for ascension of the ureters as non-flagellated bacteria were unable to reach the kidneys in the murine model of UTI [8]–[10]. Since motility is important for a successful infection, chemotaxis may also play a part in the efficient and rapid colonization of the urinary tract by directing the bacteria up the urethra and ureters.

Chemotaxis is defined as the movement toward an attractant or away from a repellant, and the mechanism behind this behavior is well characterized in non-pathogenic *E. coli* strain K-12. The chemotaxis system consists of chemoreceptors, or methyl-accepting chemotaxis proteins, that possess a variable periplasmic ligand binding domain and a conserved cytoplasmic signaling domain. When the periplasmic binding site of the chemoreceptor is occupied, a conformational change occurs that inhibits the activity of CheA, a histidine kinase that is docked to the chemoreceptor through CheW. CheA donates the phosphoryl group to CheY, a cytoplasmic protein that shuttles between the chemoreceptor complex and the flagellar motor to complete transduction of the signal from the chemoreceptor. Phosphorylated CheY binds to a component of the flagellar motor and changes the direction of flagellar rotation to produce a tumble [11], [12]. To further increase the magnitude of the response to a ligand, dimers of individual chemoreceptors form teams of mixed trimers that form large signaling clusters capable of amplifying the signal from a few chemoreceptors. Signaling through the chemoreceptors is therefore a cooperative process [13].

*E. coli* strain K-12 possesses five known chemoreceptors including Tsr (L-serine), Tar (aspartate/maltose), Trg (galactose/ribose), Tap (dipeptides) and Aer (oxygen/redox state) [11], [14]. The prototypical UPEC strain CFT073 encodes only three of the five chemoreceptors: *tsr*, *tar* and *aer*. A previous study looking at the prevalence of chemoreceptors across *E. coli* strain groups found that the loss of the Trg and Tap receptors is common among UPEC strains [15], suggesting that the remaining three chemoreceptors are useful for survival in the urinary tract. Loss of *chew* was also found to attenuate UPEC in the murine model of UTI, indicating that chemotaxis is important for a successful infection [8]. However, it is unknown which components of urine act as chemoattractants for *E. coli* and which chemoreceptors are responsible for detecting those attractants.

In this study, we show that the L-enantiomers of a subset of amino acids act as strong attractants. Other components of urine including some D-amino acids, caffeine and glucose may also act as weak attractants. We also demonstrate that deletion of any two chemoreceptors does not affect the ability of CFT073 to persist the urinary tract in the murine model. Taken together, this evidence indicates that the chemoreceptors of *E. coli* strain CFT073 may perform redundant functions in the urinary tract.

## Materials and Methods

### Ethics Statement

This study was carried out in strict accordance with the recommendations in the Guide for the Care and Use of Laboratory Animals of the National Institutes of Health. The protocol was approved by the UW-Madison Animal Care and Use Committee (Permit Number: M00450-0-07-08). All efforts were made to minimize suffering. Human urine samples were obtained from a voluntary participant who gave written consent. Permission for collection of human urine was obtained from the University of Wisconsin Health Sciences Institutional Review Board (IRB). All samples were obtained and used at the University of Wisconsin-Madison.

### Bacterial Strains and Growth Conditions

*E. coli* strain CFT073 was isolated from the blood and urine of a woman admitted to the University of Maryland Medical System with pyelonephritis [16]. *E. coli* strain RP437 was a gift from Alan Wolfe. All strains were grown in either Luria Broth (LB) or filter sterilized human female urine from a single volunteer. When growing RP437 in urine, 0.25 mg/mL of L-histidine, L-leucine and L-methionine had to be added to allow for growth. All strains were grown at 37°C with shaking at 250 rpm. Urine swim plates were made by mixing 3 parts sterile urine to 1 part sterile agar solution to a final concentration of 0.3% agar. All swim plates were incubated at 37°C.

### Construction of Strains

All gene deletions were performed using the λ-Red recombination system developed by Datsenko and Wanner [17]. Plasmids pKD3 and pKD4 were used to generate the specific λ-Red PCR products for transformation. The antibiotic resistance cassette used to replace the target gene was removed using a Flp recombinase encoded on pCP20, leaving a small, nonpolar scar sequence in place of the deleted gene [17]. All gene deletions were verified by PCR and loss of antibiotic resistance on LB agar containing the appropriate antibiotic. Primers used for PCR generation of the λ-Red fragments are listed in Table S1.

Allelic repair of the *tsr*, *tar* and *aer* deletion mutants were constructed by transducing each chemoreceptor gene linked to a kanamycin marker outside of the targeted operon into its corresponding deletion mutant using the *E. coli* CFT073 transducing phage EB49 (8). The marker was removed via electroporation of the transductants with pCP20. Insertion of each gene was verified by PCR and sequencing of the chemoreceptor gene.

### Capillary Chemotaxis Assay

Capillary assays were performed according to Adler’s original method [18] with a few modifications. Briefly, strains were grown overnight in urine swim plates (0.3% agar). The following morning, the outer ring of the swim colony was aseptically collected and incubated for a further 3–4 hours in 8 mL urine at 37°C with shaking. Bacteria were then harvested and washed once in chemotaxis buffer (10^−2^ M phosphate buffer, 10^−4^ M EDTA in ddH_2_0). Bacteria were resuspended to OD_600_∼0.387 in chemotaxis buffer. Capillary tubes (1 mm) were sealed on one end and filled with 0.5 ul of the attractant solution at the indicated concentrations. Buffer alone was used as a control for random motility and undiluted urine or 500 µg/mL L-aspartate were used as positive controls for all assays. Capillary tubes were then incubated with 100 µl of the bacterial suspension at 30°C for 45 minutes. Capillary tubes were washed and the contents were dilution plated. Reported values for each strain and attractant represent combined data from at least three independent assays. Response levels above the buffer control were graphed and analyzed using a two-way ANOVA and Bonferroni post tests with Prism (GraphPad).

### Murine Model of UTI

Colonization of the urinary tract was determined using the competitive murine model of urinary tract infection as described previously [19]. CFT073Δ*lacZYA* was used as the wild-type strain and the chemotaxis mutants had an intact *lacZYA* locus. To select for piliated bacteria, all bacterial strains were grown statically in 3 mL LB at 37°C for 2 days. The pellicle formed on the rim of the test tube was then passaged to fresh LB, incubated for 2 more days, and finally passaged again to 40 mL LB for a final 2 day incubation. The broths were adjusted to OD_600_∼0.4 with 1×PBS and the wild-type strain and the mutant strain were mixed equally. The mixed broth was then centrifuged and the cell pellet washed once in 1×PBS and resuspended in 500 µl 1×PBS. Isofluorane-anesthetized 6–7 week-old female Swiss Webster mice (Harlan, USA) were inoculated via urethral catheterization with 50 µl (10^8^ CFU) of the mixed bacterial suspension. Mice were euthanized via CO_2_ asphyxiation and the bladder and kidneys were aseptically harvested and placed in 1×PBS. The organs were homogenized, serially diluted in 1×PBS and plated on MacConkey agar medium (Fisher, USA). Surviving strain ratios were determined by counting white (wild-type) and red (mutant) colonies. Colonization levels were graphed and analyzed using a paired Wilcoxon signed ranked test and Prism (GraphPad).

## Results

### Chemotaxis Toward Human Urine is Mainly Mediated by Tsr and Tar in *E. coli* CFT073

**Figure 1:**
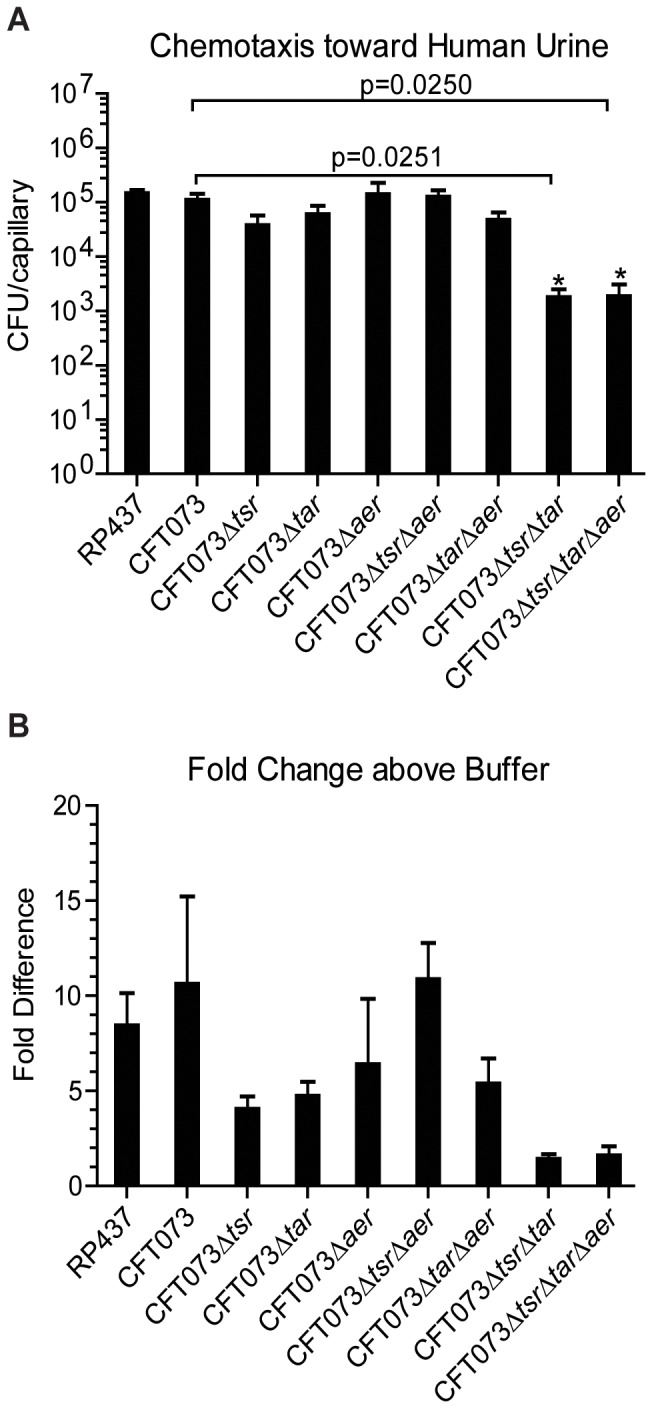
Chemotaxis of wild-type CFT073 and CFT073 chemoreceptor mutants toward undiluted human urine. The colony forming units (CFUs) normalized to the buffer control (A) and the fold increase relative to the buffer control (B) are shown for each strain. Each strain was tested in triplicate, and a nonparametric T-test used to calculate statistical significance between the wild-type and mutant strains.

While it is known that *E. coli* CFT073 displays chemotaxis toward urine [15], it was unclear if all three chemoreceptors were involved in mediating this attraction. To determine if one or all chemoreceptors are involved in chemotaxis toward urine, a series of chemoreceptor mutants with all combinations of the genes encoding Tsr, Tar and Aer were deleted from the CFT073 genome via the λ-Red system [17]. Each mutant strain was then tested in the capillary assay for chemotaxis toward undiluted human urine and compared to the wild-type CFT073 response. Since CFT073, unlike K-12, does not flagellate well when grown in broth culture, the bacteria were first grown in swim plates and then transferred to broth culture for all capillary assays. The results showed that Tsr and Tar are capable of mediating chemotaxis toward urine when present alone. When Aer is the only chemoreceptor present, however, chemotaxis toward urine is almost completely abolished (Fig. 1). These results suggest that Tsr and Tar mediate most of the observed attraction toward urine.

### Physiological Levels of a Subset of L-amino Acids are Chemoattractants for *E. coli* CFT073

As was first reported by J. Adler, *E. coli* K-12 is strongly attracted to a variety of L-amino acids [14]. All 22 L-amino acids are excreted in human urine, although some are found in higher concentrations than others [20]–[23]. To determine which of these amino acids could act as attractants for CFT073 during infection, each individual amino acid at its upper physiological concentration found in human urine was used as the attractant for wild-type CFT073 in the capillary assay. As was previously reported by Lane et al. [15], aspartate is an attractant for CFT073. We also identified six other amino acids that act as strong attractants for CFT073 including alanine, asparagine, cysteine, glutamate, glycine and serine. These results concur with the earlier research by J. Adler done in *E. coli* K-12 strain AW405 [14](Table 1). Since the responses at the high concentrations found in urine were low, serine and aspartate were tested at alternate concentrations that are comparable to the peak concentrations used in Adler’s studies (1 mg/mL and 500 µg/mL, respectively) to verify that these amino acids do act as attractants (Table 1). Capillary assay results for K-12 strain RP437 cells prepared in the same way as CFT073 are shown in Table 1 to illustrate the similarity of responses to attractants between the two strains.

**Table 1:** Strong attractants for *E. coli* CFT073.

Component^e^	Reported HighValue in Urine(mg/mL)^f^	ConcentrationTested (mg/mL)	CFT073 CFU Accumulated(×10^3^)a,b,c	CFT073 FoldChange to Buffer^a^	RP437 CFU Accumulated (×10^3^)a,b,c
L-Ala	6.2	5	284±150^c^	10.7±4.8	256±87^c^
L-Asn	2.9	3	211±130^c^	9.8±5.1	104±27^c^
L-Asp	1.2	1	62±51	2.7±1.2	86±19^c^
		0.5	209±68^c^	8.1±1.4	192±24^c^
L-Cys	4.0	1	320±154^c^	15.1±8.6	312±87^c^
L-Glu	1.7	1.5	157±108^c^	6.3±2.4	130±17^c^
L-Gly	18.0	10	161±87^c^	6.8±2.5	167±43^c^
L-Ser	7.3	5	15±10	1.6±0.4	135±61^c^
		1	249±42^c^	9.0±1.3	222±35^c^

a Values are means ± standard deviations (n = 3–6).

b Normalized to buffer control.

c Statistical significance (p<0.05) from buffer control was calculated using a 2-way ANOVA analysis with Bonferroni post-tests on CFU/capillary data.

d BB = Below buffer.

e Responses toward arginine, cystine, glutamine, isoleucine, leucine, lysine, phenylalanine, proline, taurine, tryptophan, tyrosine, and valine were all below the buffer control.

f See references 20–23.

The chemoreceptor(s) responsible for sensing each individual L-amino acid were investigated using the chemoreceptor knock out mutants in the capillary assay. These experimental results are similar to previously published reports with *E. coli* K-12 with regards to amino acids sensed by Tsr and Tar [14]. The *tar* deletion mutant showed decreased chemotaxis toward asparagine, aspartate and glutamate and loss of either *tsr* or *tar* resulted in a decreased response toward alanine, cysteine, glycine and serine. Our results indicate that loss of *aer* also lowers the chemotactic response toward alanine and cysteine (Table 2). Allelic repair via transduction with the appropriate chemoreceptor gene for each mutant strain restored the altered responses of all of the L-amino acids to wild-type levels (data not shown).

**Table 2:** Chemotaxis by *E. coli* CFT073 receptor mutants toward tested components.

Component	Conc. Tested (mg/mL)	Fold Change to Buffer^a^				Allelic Repair
		Wild-type	Δ*tsr*	Δ*tar*	Δ*aer*	
L-Ser	1	9.0±1.3	**1.0±0.01** b	2.7±1.3	6.7±2.2	+
L-Gly	10	6.8±2.5	**1.0±0.1** b	1.5±0.9	5.0±2.7	+
L-Asn	3	9.8±5.1	5.9±4.2	**2.5±2.1** b	7.4±5.1	+
L-Asp	1	8.1±1.4	6.7±1.1	**0.5±0.2** b	5.4±1.1	+
L-Glu	1.5	6.3±2.4	9.7±6.9	**0.4±0.2** b	4.4±4.7	+
L-Ala	5	10.7±4.8	**1.2±0.2** b	**1.7±1.0** b	**4.3±4.4** b	+
L-Cys	1	15.1±8.6	**2.8±1.8** b	**2.2±1.5** b	**6.4±4.7** b	+
L-His	20	1.4±0.2	2.8±2.5	0.7±0.5	2.1±0.8	+
L-Met	15	2.9±0.8	3.3±1.4	0.8±0.2	1.6±0.4	+
L-Thr	0.12	1.4±0.2	0.6±0.1	1.8±0.7	1.9±0.4	+
D-Ala	0.5	2.8±1.8	0.7±0.1	1.5±0.7	1.6±0.3	+
D-Asn	0.3	3.9±2.1	8.3±3.7	1.2±0.7	2.0±0.3	+
D-Ser	0.2	5.8±4.6	0.9±0.1	1.7±0.9	3.1±1.3	+
D-Phe	0.004	1.4±0.3	1.0±0.3	0.7±0.3	1.8±0.2	-
D-Pro	0.002	1.5±0.6	0.9±0.2	0.6±0.3	0.9±0.5	-
D-Thr	0.0014	1.4±0.3	0.9±0.2	0.6±0.2	1.0±0.6	-
D-Tyr	0.0025	1.3±0.3	0.8±0.3	0.6±0.2	1.1±0.6	-
Caffeine	0.02	1.1±0.2	0.96±0.2	1.1±0.1	1.1±0.1	-

a Values are means ± standard deviations (n = 3–6).

b Statistical significance (p<0.05) from wild-type CFT073 was calculated using a 2-way ANOVA analysis with Bonferroni post-tests using CFU/capillary above buffer data.

### Some L- and D-amino Acids may Elicit a Weak Chemotactic Response from *E. coli* CFT073

L-methionine, L-histidine and L-threonine were also tested in the capillary assay and accumulated a few bacteria above the buffer only control. However, the results for L-histidine and L-threonine were not statistically significant and are likely not biologically significant (Table 3). Methionine was also tested at the concentration used by Adler (15 mg/mL). The altered concentration increased the total accumulated bacteria (Table 3). This indicates that L-methionine may act as an attractant for CFT073 although the results were still not statistically significant. The results for all other L-amino acids were below the buffer only control. Deletion of *tar* appeared to decrease the number of bacteria accumulated for L-methionine and L-histidine, and deletion of *tsr* decreased accumulation for L-threonine (Table 2). However, these results were not statistically significant and it is not clear if these L-amino acids elicit a chemotactic response from *E. coli* CFT073.

**Table 3:** Possible weak attractants and non-attractants for *E. coli* CFT073.

Component	Reported HighValue in Urine(µg/mL)^f^	ConcentrationTested (µg/mL)	CFT073 CFUAccumulated (×10^3^)a,b,c	CFT073 FoldChange to Buffer^a^	RP437 CFU Accumulated (×10^3^)a,b,c,d
L-Met	2.3	2	5±14	1.1±0.4	12±5
		15	58±29	2.9±0.8	60±9
D-Ala	4.79	500	60±71	2.8±1.8	23±8
		10	2±3	1.0±0.6	NT
D-Ser	114	200	96±68	5.8±4.6	10±2
		100	17±8	2.8±1.2	NT
D-Asn	0.1189	300	92±68	3.9±2.1	178±43^e^
		0.3	BB		NT
L-His	23300	20000	10±2	1.4±0.2	BB
L-Thr	3800	120	8±3	1.4±0.2	6±1
D-Phe	2.04	4	4±3	1.4±0.3	BB
		2	4±2	1.4±0.4	NT
D-Pro	1.34	2	5±3	1.5±0.6	BB
		1	3±4	1.2±0.4	NT
D-Thr	0.69	1.4	3±1	1.4±0.3	11±14
		0.7	4±5	1.3±0.5	NT
D-Tyr	2.51	5	BB		BB
		2.5	3±4	1.3±0.4	NT
Caffeine	10.26	20	2±3	1.1±0.2	BB
		5	4±2	1.2±0.1	NT
D-glucose	200	250	3±9	1.4±0.6	NT
		125	0.3±9	1.3±0.7	NT

a Values are means ± standard deviations (n = 3–6).

b Normalized to buffer control.

c BB = Below buffer.

d NT = Not tested.

e Statistical significance (p<0.05) from buffer control was calculated using a 2-way ANOVA analysis with Bonferroni post-tests on CFU/capillary data.

f See references 20–30.

In addition to L-amino acids, some D-amino acids (D-alanine, D-asparagine, D-phenylalanine, D-proline, D-serine, D-threonine, D-tryptophan, D-tyrosine, and D-valine) are also found in urine with D-serine and D-alanine present in the highest concentrations [24]–[27]. These D-amino acids were tested at their physiological concentrations with wild-type CFT073 in the capillary assay to determine if any were attractants. D-asparagine, D-tryptophan and D-valine were the only D-amino acids tested that did not show a response above the buffer control. Physiological concentrations of D-alanine, D-phenylalanine, D-proline, D-threonine, and D-tyrosine all produced a negligible response while D-serine produced a weak response (Table 3). Adler also noted a positive response toward D-serine by K-12 in his report (33).

Since D-serine is present in much higher concentrations in urine than any of the other D-amino acids, the greater effect observed may be due to the higher concentration tested and not to a specific preference for D-serine by CFT073. Therefore, all D-amino acids were tested at 300–500 µg/mL to determine if a concentration increase alone could amplify the bacterial accumulation in the capillary tubes loaded with each D-amino acid. When tested at the higher concentration, the accumulation using D-alanine and D-asparagine increased when compared to their physiological concentrations (Table 3). D-phenylalanine, D-proline, D-threonine, D-tryptophan, D-tyrosine, and D-valine did not produce a response above buffer at the higher concentration (data not shown). The results obtained for K-12 strain RP437 toward the D-amino acids again displays the similarity of response to attractants between the two strains (Table 3). These data indicate that D-amino acids, in addition to L-amino acids, may be sensed by the chemosensory machinery of CFT073 and may influence the chemotactic behavior of *E. coli in vivo*.

The chemoreceptor(s) responsible for the response to the D-amino acids were determined using the chemoreceptor mutants with the amino acid concentration that elicited the greatest response. Loss of *tsr* diminished the response to D-alanine, D-phenylalanine, D-proline, D-serine, D-threonine, and D-tyrosine. Loss of *tar* lessened the response toward D-alanine, D-asparagine, D-phenylalanine, D-proline, D-serine, D-threonine, and D-tyrosine while the loss of *aer* partially diminished chemotaxis toward D-alanine, D-asparagine, D-phenylalanine, D-proline and D-tyrosine (Table 2). The strains with allelic repair of the chemoreceptor genes were tested in the capillary assay for a return to the wild-type phenotype. Unlike the results for the L-amino acids, the repaired *tsr* strain showed a return to wild-type response levels for only D-alanine and D-serine, and the repaired *tar* strain responded like the wild-type strain to just D-alanine, D-asparagine and D-serine. The repaired *aer* strain did not return the response of any of the D-amino acids tested to wild-type levels (data not shown).

### Most Non-amino Acid Components of Urine do not Act as Chemoattractants for *E. coli* CFT073

A large portion of the solutes in human urine consists of organic compounds in addition to amino acids [28]–[31]. We tested a large subset of the compounds found in the highest abundance in human urine as there were no reports of their study as chemoattractants. Urea, creatine, creatinine, glucuronic acid, uric acid, imidazole, glucose, inositol, urobilin, allantoin, 2′-deoxyadenosine 5′-triphosphate, 2′-deoxyguanosine 5′-triphosphate and caffeine were tested in the capillary assay at the higher range of their physiological concentrations. As reported previously in K-12 [32], glucose elicited a response from wild-type CFT073, but this response was much weaker than observed for K-12 at the tested concentration. Caffeine also produced slight accumulation of wild-type CFT073 that was not statistically significant and likely not biologically significant (Table 3). No other component tested produced a response above the buffer baseline (data not shown). When caffeine was tested as an attractant with the chemoreceptor knock out mutants, only the *tsr* mutant displayed a decrease in its response (Table 2). However, allelic repair of the *tsr* mutant did not bring the response to caffeine back up to wild-type levels (data not shown). Because it has been determined that glucose is detected through the interaction of unphosphorylated Enzyme I of the phosphoenolpyruvate-dependent carbohydrate phosphotransferase system with the chemotaxis machinery [32]–[35], the chemoreceptor mutants were not tested with glucose as an attractant.

A variety of hormones are also secreted in urine in the ng/mL range, including dopamine, norepinephrine, epinephrine, estrogens and androsterone [36]–[39]. Another laboratory has shown that enterohemorrhagic *E. coli* is attracted to norepinephrine and epinephrine [40]. To determine if CFT073 is also attracted to hormones, dopamine, norepinephrine, epinephrine, estradiol, estrone, estriol and androsterone were tested as potential attractants for CFT073 in the capillary assay. Unlike enterohemorrhagic *E. coli*, CFT073 did not respond to epinephrine, norepinephrine or any of the other hormones at their physiological concentrations (data not shown). Therefore, hormones are unlikely to act as attractants for uropathogenic *E. coli* in the urinary tract.

### Loss of Any Two Chemoreceptors does not Affect Survival *in vivo*

**Figure 2:**
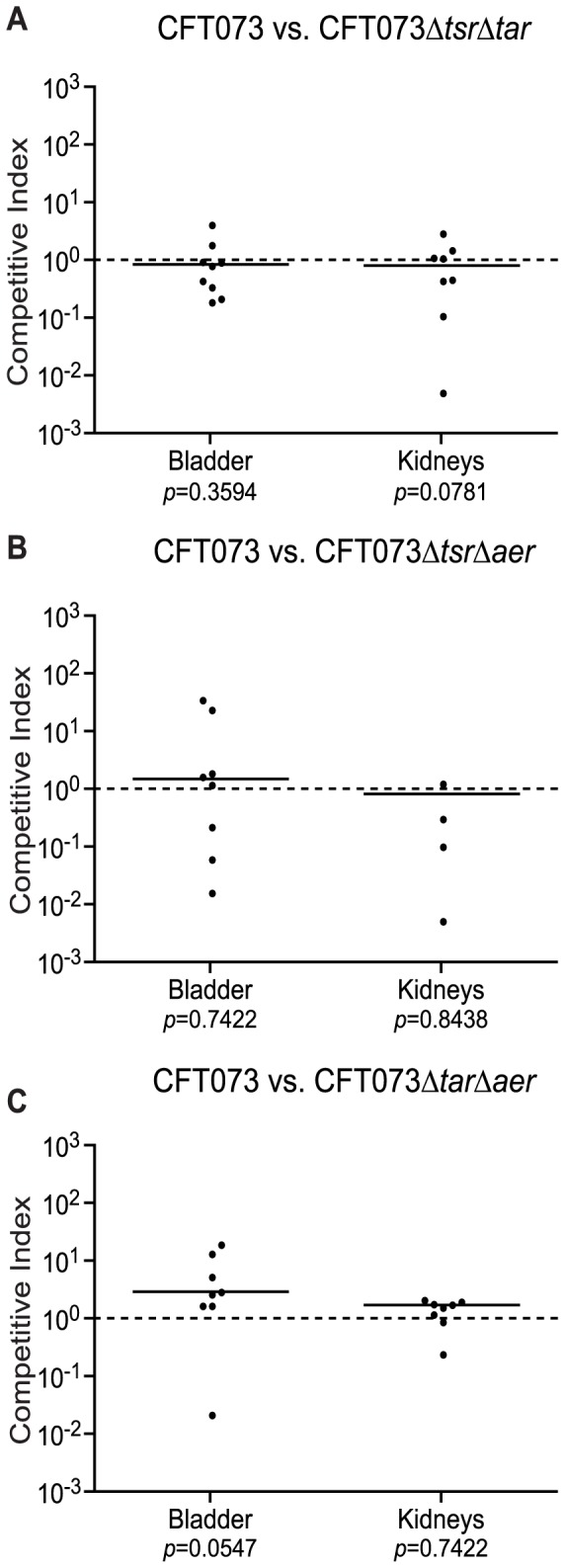
Chemoreceptor mutants compete like the wild-type strain *in vivo*. The results for competitive co-infection with wild-type CFT073 and CFT073ΔtsrΔtar (n = 9) (A), CFT073ΔtsrΔaer (n = 8) (B), CFT073ΔtarΔaer (n = 8) (C) in the mouse model of UTI are shown. The competitive indices were calculated by the following equation: (mutant CFU recovered/wild-type CFU recovered)/(mutant inoculum CFU/wild-type inoculum CFU). The line indicates the median value.

Studies with *Vibrio cholerae*, *Helicobacter pylori* and *Campylobacter jejuni* reported the necessity for chemotaxis in the colonization of each pathogens’ infectious niche [41]–[45]. To determine if any one chemoreceptor is important for colonization of the urinary tract, female Swiss-Webster mice were co-infected with an equal mix of wild-type CFT073 and a CFT073 double chemoreceptor deletion mutant via urethral catheterization. All animals were sacrificed at 48 hours post-infection. The ratios of the wild-type strain to the mutant strain recovered from the bladder and the kidneys revealed that the loss of any two chemoreceptors did not affect the ability of the mutant strain to compete with the wild-type strain (Fig. 2 A–C).

## Discussion

Initial events in the colonization of the urinary tract by UPEC remain to be characterized in detail. We hypothesize that navigation to and in the urinary tract via chemotaxis are among the first steps to aid UPEC colonization of the urethra and bladder. As previously shown, uropathogenic *E. coli* CFT073 is attracted to undiluted human urine, demonstrating that the attraction of CFT073 to urine may aid the bacteria in gaining access to the urinary tract [15]. In support of this hypothesis, other bacterial species use chemotaxis to direct colonization of specific regions of the digestive tract (e.g. *Vibrio cholerae* to the deep crypts of the small intestine and *Campylobacter jejuni* to the mucus of the gastric epithelium) [41], [42], [44]. Additionally, the plant pathogen *Ralstonia solanacearum* uses aerotaxis to quickly find the roots of its host plant to initiate colonization [43], [46]. Multiple species of bacteria use chemotaxis to direct colonization to their preferred niches, highlighting chemotaxis as an important colonization factor during pathogenesis.

We sought to determine which chemoreceptor(s) is responsible for the attraction of CFT073 to urine. CFT073 deletion mutants missing one of the three chemoreceptors (Δ*tsr*, Δ*tar*, or Δ*aer*) were subsequently tested for chemotaxis toward human urine. Loss of any one chemoreceptor did not result in significant loss of chemotaxis toward urine, indicating that there were redundant ligands shared between the chemoreceptors and/or that there were multiple strong attractants in urine that are sensed by different chemoreceptors. When both *tsr* and *tar* were absent, the positive response to urine was almost abolished, indicating that these two chemoreceptors are mainly responsible for chemotaxis toward urine. The loss of *aer* did not change the response to urine to a significant degree. One explanation for the loss of a response toward urine when only *aer* is present may be that the small number of Aer receptors, which is markedly lower than Tsr and Tar levels [47], is simply too small to direct an efficient chemotactic response without the aid of Tsr and Tar to form the receptor clusters [48]. Therefore, urine could still stimulate a response through Aer, but the resulting signal would be too small to effect a chemotactic response that could be detected by the capillary assay. Aer could also be important for indirectly sensing oxygen within the urinary tract, but aerotaxis was not tested in this study.

The results obtained in this study with *E. coli* CFT073 mostly concur with the results obtained previously with *E. coli* K-12. Just as with K-12, CFT073 is attracted to a variety of L-amino acids including alanine, asparagine, aspartate, cysteine, glutamate, glycine and serine. Methionine, threonine and histidine also stimulated accumulation of the bacteria in the capillary tubes, but these responses were not statistically significant. The apparent dose response to L-methionine indicates that this amino acid may be a weak attractant for CFT073 despite the lack of statistical significance. The responses obtained in this report were also slightly lower than in Adler’s original report, a difference that might be accounted for by growth in urine instead of MOPS glycerol and some slight differences in the purity of the attractants used. The increased response of CFT073 toward lower concentrations of L-serine and L-aspartate demonstrates that an optimum concentration of each attractant exists that will stimulate chemotaxis. Concentrations over that optimum may saturate the receptors and decrease chemotactic behavior. Therefore, the optimum concentration of attractants in the urine of an individual may be necessary to maximize the ability of the bacteria to ascend the urinary tract. The optimum concentrations for L-aspartate and L-serine are below the highest reported concentrations of each amino acid in human urine. This suggests that concentrated urine with the highest levels of L-aspartate and L-serine may dampen the chemotactic response of CFT073.

The chemoreceptor mutant experiments to determine whether Tsr or Tar mediates the response to each L-amino acid did agree with previous reports [14]. The chemoreceptor deletion mutants also illustrate that the chemoreceptors are needed to see the response to each attractant, indicating that the accumulation of bacteria detected in the capillary tubes is due to a true chemotactic response. Accumulation for a few of the attractants was still detected after the deletion of *tsr* or *tar*, a result that could indicate that both Tsr and Tar are capable of sensing the particular attractant or that the particular attractant stimulates chemokinesis as well as chemotaxis. Additionally, this study identified Aer as a partial mediator of chemotaxis toward L-alanine and L-cysteine in *E. coli* CFT073.

In addition to the response to L-amino acids, this study also identified several D-amino acids and two non-amino acid components of urine that produced accumulation of *E. coli* CFT073 above buffer within their physiological ranges. However, the results for these components were not statistically significant above buffer and may not be truly capable of eliciting a response through the chemoreceptors. These components include D-alanine, D-phenylalanine, D-proline, D-serine, D-threonine, D-tyrosine, glucose and caffeine with D-serine exhibiting the strongest response. The other D-amino acids on this list as well as glucose and caffeine weakly responded even at increased concentrations, although D-asparagine generated a response that surpassed that of D-serine when tested at a concentration that far exceeded its reported concentration range in urine. Since concentrations of each amino acid can vary widely from person to person, the reported range of D-asparagine in human urine is by no means absolute. Therefore, D-asparagine may act as an attractant in urine for UPEC when its concentration is sufficiently high. Capillary assays using the mutant strains with allelic repair of the deleted chemoreceptor gene showed that only the responses toward D-alanine, D-asparagine and D-serine could be returned to wild-type levels. This suggests that, from the above group, only these three D-amino acids may elicit a true response through the chemoreceptors.

Our results also indicate that loss of any two of the chemoreceptors does not affect colonization of the urinary tract in the mouse model. However, the direct inoculation of the bladder and potential reflux of bacteria into the kidneys of the mice immediately after inoculation prevents any conclusions that can be made about the necessity of chemotaxis for ascension of the urethra and ureters and could mask any potential colonization defects. A better model for ascension of the urinary tract is needed to address this issue. Additionally, the chemotaxis system could be important for not just ascension of the urinary tract but penetration of the mucus layer to achieve close contact with the bladder epithelium [49], [50]. Without the ability to switch rotation of the flagella and the direction of net movement, the bacteria could become stuck in the mucus layer leading to eventual expulsion of the bacteria from the urinary tract by micturition. One final curious observation is that, while the CFT073Δ*tsr*Δ*tar* mutant was attenuated for chemotaxis toward urine *in vitro*, this strain performed like wild-type CFT073 in the mouse model. Although Aer protein levels appear insufficient to produce a detectable response in the capillary assay, they may be sufficient to produce a chemotactic response or switching of the flagellar motor that keeps the bacteria competitive during infection. Reflux during inoculation may also conceal any defects that may be observed *in vivo* using the CFT073Δ*tsr*Δ*tar* mutant.

This study has shown that amino acids, possibly D-forms as well as L-forms, act as attractants for *E. coli* CFT073 within their physiological concentrations in urine and that Tsr and Tar are primarily responsible for sensing these attractants. Loss of any two chemoreceptors did not result in a fitness defect *in vivo*, although the current mouse model may be a poor tool to evaluate the contribution of each chemoreceptor in the infectious process. Despite this limitation, a possible set of ascension steps in which chemotaxis may play a role can be imagined. Ascension aided by chemotaxis from the relatively nutrient poor region of the urethral opening to the bladder may be stimulated by exposure to urine during voiding of the bladder. The short female urethra may also allow the bacteria to gain access to the bladder as a result of physical trauma to the urethral area. Ascension from the bladder to the kidneys may occur after increasing numbers of growing bacteria in the bladder consume most of the available nutrients, thus creating a gradient from the bladder up the ureters. This gradient may then stimulate chemotaxis and enhanced movement up the ureters to the kidneys. The ability to navigate the urinary tract may result in more efficient colonization, lessening the possibility that the bacteria would be dispelled by urine before being able to colonize the bladder. An improved method for monitoring early events in the colonization of the urinary tract are needed to determine if chemotaxis plays a part in the ascension of the urethra and ureters to colonize the urinary tract.

## Supporting Information

Table S1 **Primers used in this study.**
(DOCX)
