# Supplementary material for: Chemoreceptors of Escherichia coli CFT073 Play Redundant Roles in Chemotaxis toward Urine
Source: PLoS One. 2013 Jan 30;8(1):e54133. doi: 10.1371/journal.pone.0054133 (PMC3559539; doi:10.1371/journal.pone.0054133)
Supplement: Table S1 — Primers used in this study. (DOCX) [file pone.0054133.s001.docx]

| **Gene/Application** | **Sequence (5’-3’)** |
| --- | --- |
| *tsr* deletion | GGCCGAAAATCTTGCATCGGTCCACAGGAAAGAGAAACCTGTGTAGGCTGGAGCTGCTTC |
|  | AAAACGCCGGATGAAATACTCATCCGGCATCATTACGCATATGAATATCCTCCTTAG |
| *tsr* deletion check | CGAGAACCGGACCCACCAG |
|  | CCTGGCCTGCGCTGTTCC |
| *tar* deletion | CCCATCAGGCGGCAATGACCGCTTTAGTAAATACTCGTGTGTAGGCTGGAGCTGCTTCG |
|  | AATAAAGTTTCCCCCCTCCTTGCCGATAACGAGATCAACCATATGAATATCCTCCTTAG |
| *tar* deletion check | CCAATGTATCAGCCAGCGTC |
|  | CTACGCCTTGCTGGTGGATC |
| *aer* deletion | GGCATTGTGCTCCAACCGCTGGATCCGGCATACCGATGTGTAGGCTGGAGCTGCTTCG |
|  | GAAGTTAACAACCATATAACCTGCACAGGACGCGAACCATATGAATATCCTCCTTAG |
| *aer* deletion check | CGATGACAGCCACGGTTACG |
|  | GCTTGCCACTCTACGGCTC |
| *φtsr* (*tsr*-*c5431*) | ACGTAATGATGCCGGATGAGTATTTCATCCGGCGTTTTTGTGTAGGCTGGAGCTGCTTCG |
|  | ATATCCCGGCGATTTCACTGTTCCTGGTTTAATAATAAACCATATGAATATCCTCCTTAG |
| *φtar* (*argS*-*flhE*) | AGCGTATGTAATCGCGTTATACGGCAACAGCCGATGTTTGTGTAGGCTGGAGCTGCTTCG |
|  | AATTATCGTTAATGATAAAAAAGCCGATGTCTGCAATAACCATATGAATATCCTCCTTAG |
| *φaer*(*aer*-*ygiL*) | ATATTACGCAACTGGATTAATCGCCGCATCCGCCAGTGTGTGTAGGCTGGAGCTGCTTCG |
|  | GGCGCGTCAAGCGTCGCATCCGGCAATTGCACCGCGCAACCATATGAATATCCTCCTTAG |
